# Serum CXCL8 as a biomarker for predicting ALNM in breast cancer: Combined diagnostic value with tumor markers and ultrasound

**DOI:** 10.5937/jomb0-58742

**Published:** 2025-11-05

**Authors:** Jinxiang Hou, Ceng Li

**Affiliations:** 1 Xuzhou Maternal and Child Health Hospital, Department of Ultrasound, Xuzhou, Jiangsu, 221009, China; 2 Xuzhou Cancer Hospital, Department of Radiology and PET/CT Centre, Xuzhou New Healthy Geriatric Hospital, Xuzhou, Jiangsu, 221009, China

**Keywords:** breast cancer, lymphatic node transfer, CXCL8, tumour marker, diagnostic biomarker, ultrasound imaging, karcinom dojke, metastaza u limfnim čvorovima, CXCL8, tumorski marker, dijagnostički biomarker, ultrazvučno snimanje

## Abstract

**Background:**

This study analysed the relationship between serum chemokine CXC ligand 8 (CXCL8) and axillary lymph node metastasis (ALNM) in breast cancer (BC), evaluating its predictive value when combined with tumour markers and ultrasound imaging.

**Methods:**

121 BC patients and 104 healthy controls were included, and serum CXCL8 was detected by enzyme-linked immunosorbent assay (ELISA) to compare the differences in the levels of CXCL8 and tumour markers in the two study groups. Pathological examinations revealed that 36 of the patients had ALNM. To further evaluate the diagnostic value, the receiver operating characteristic (ROC) curve was employed to analyse the ability of CXCL8, tumour marker and combined colour Doppler ultrasound blood flow richness grade (Adler grade) to assess ALNM in BC patients.

**Results:**

Serum CXCL8 carcinoembryonic antigen (CEA), carbohydrate antigen (CA) 153, and CA27.29 were higher in BC patients than in controls (P&lt; 0.05). Patients with ALNM had higher levels of CXCL8, CEA, CA153, and CA27.29 (P&lt; 0.05). The combined model (CXCL8 + tumour markers + Adler grade) achieved an AUC of 0.903 (95% CI: 0 .8 5 0 -0 .9 5 7 ), with 86.11% sensitivity and 82.35% specificity (P&lt; 0.001).

**Conclusions:**

High expression of CXCL8 is closely associated with BC ALNM.

## Abstract (sr)

**Uvod:**

Ova studija je analizirala povezanost između serumske koncentracije hemokina CXC liganda 8 (CXCL8) i metastaza u aksilarnim limfnim čvorovima (ALNM) kod karcinoma dojke (BC), procenjujući njegovu prediktivnu vrednost u kombinaciji sa tumorskim markerima i ultrazvučnim snimanjem.

**Metode:**

U istraživanje je bila uključena 121 pacijentkinja sa karcinomom dojke i 104 zdravih kontrolnih ispitanica. Nivoi CXCL8 u serumu su određeni pomoću ELISA metode, kako bi se uporedile razlike u nivoima CXCL8 i tumorskim markerima između dve grupe. Patoloskim pregledom je utvrđeno da 36 pacijentkinja ima ALNM. Da bi se dodatno procenila dijagnostička vrednost, korisćena je ROC kriva za analizu sposobnosti CXCL8, tumorskih markera i kombinovanog Dopler ultrazvučnog stepena vaskularizacije (Adlerova skala) u proceni ALNM kod pacijentkinja sa BC.

**Rezultati:**

Nivoi serumske koncentracije CXCL8, karcinoembrionalnog antigena (CEA), karbohidratnog antigena (CA) 153 i CA27.29 bili su visi kod pacijentkinja sa karcinomom dojke nego kod kontrolnih pacijentkinja (P&lt; 0,05). Pacijentkinje sa ALNM su imale značajno vise nivoe CXCL8, CEA, CA153 i CA27.29 (P&lt; 0,05). Kombinovani model (CXCL8 + tumorski markeri + Adlerova skala) je postigao AUC od 0,903 (95% CI: 0,850 -0,957), sa osetljivošću od 86,11% i specifičnošću od 82,35% (P&lt; 0,001).

**Zaključak:**

Visoka ekspresija CXCL8 je usko povezana sa prisustvom ALNM kod karcinoma dojke.

## Introduction

Breast cancer (BC) ranks among the malignancies with the highest incidence in clinical practice. According to the latest global cancer burden data statistics, the number of newly diagnosed BC cases globally exceeded 2.26 million in 2020 [1]. Since the 21st century, BC has shown a steady increase in its incidence and has now overtaken lung cancer to claim the position of the most prevalent cancer worldwide [2]. However, the pathogenesis of BC has not yet been fully elucidated, and there persists a dearth of accurate and efficient early screening protocols in clinical practice [3], contributing to a rather dismal prognosis for BC patients. As a highly malignant and invasive cancer, BC ranks second only to ovarian cancer in terms of mortality among female patients [4]. A study conducted by Sun K et al. [5] revealed that the mortality rate of BC in China in 2022 was approximately 6.10 per 100,000, with the risk of death among female patients aged 55 and above being 8 times higher than that of those under. Consequently, the prevention of the onset and progression of BC has emerged as a pressing global public health concern. Meanwhile, it has become a hot spot in contemporary clinical research, attracting substantial attention from the medical community and driving continuous efforts to explore innovative preventive and therapeutic strategies [6].

In recent years, inflammatory chemokines in the tumour microenvironment have been found to be closely related to the biological behaviour of BC. Among them, chemokine CXC ligand 8 (CXCL8) promotes the proliferation and migration of tumour vascular endothelial cells by activating CXCR1/2 receptors, and its high expression is significantly correlated with aggressive subtypes of BC (e.g. triple-negative breast cancer) and distant metastasis [7]. In a recent review report, Mishra A et al. [8] concluded that CXCL8 plays a crucial role in the progression and metastasis of BC. Meanwhile, Tang S et al.'s [9] study even confirmed that elevated CXCL8 is associated with poor prognosis in triple-negative breast cancer. At the same time, some studies have found that CXCL8 targeted therapies (such as CXCR1/2 inhibitors) significantly inhibit BC metastasis in animal models [10], suggesting its clinical translational potential. Thus, it can be seen that CXCL8 has great research value in BC and is expected to become a new clinical objective assessment index for BC. However, we found that there are no studies analysing the relationship between CXCL8 and lymph node metastasis (ALNM) in BC.

Based on the research gap of CXCL8 and BC ALNM, the present study will remedy the limitations of the current study by retrospectively analysing the expression of CXCL8 in the peripheral blood of BC patients admitted to our hospital. Second, by integrating the molecular information of CXCL8 with colour Doppler ultrasound blood flow richness grade (Adler grade) [11], it is expected to break through the bottleneck of the existing ALNM prediction technology and provide a more accurate basis for individualised treatment decisions. This strategy not only deepens the understanding of the metastatic mechanism of BC but also opens up a new path for the clinical translation of a multidisciplinary joint diagnostic model.

## Materials and methods

### Study subjects

**Figure 1:**
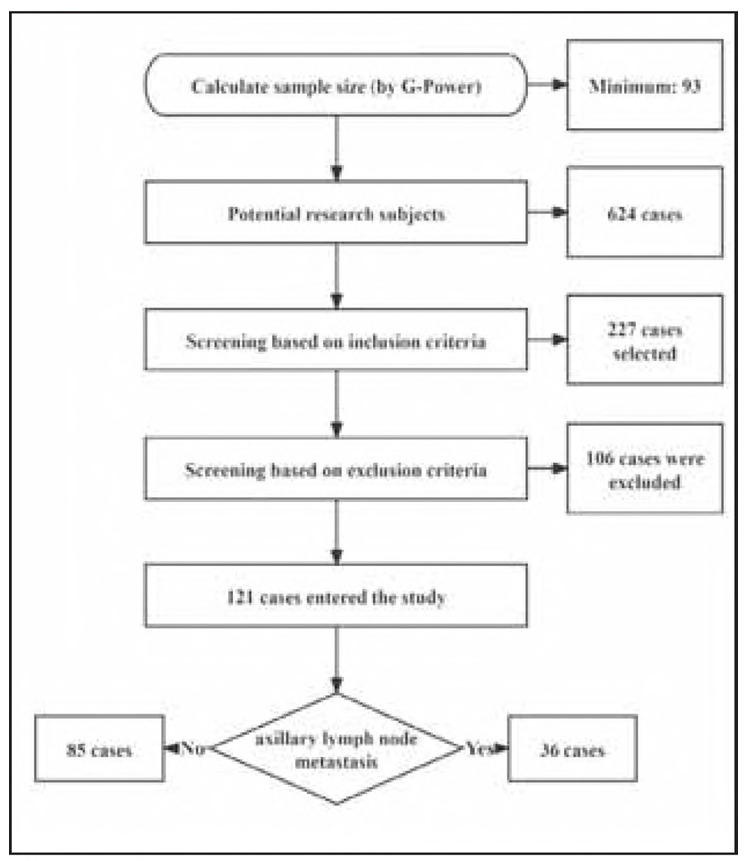
Screening process for research subjects.

We designed a retrospective analysis, with potential subjects being 624 BC patients admitted to our hospital from March 2023 to August 2024. Subsequently, G-Power software was used to calculate the minimum sample size required for this study [the G-Power calculation was based on effect size d = 0.8 (Cohen's criterion), α = 0.05, power (1-β) = 0.9, and the minimum sample size was calculated as 93 cases], and inclusion/exclusion criteria were established for screening (Figure 1). After a rigorous screening procedure, 121 BC patients were included in the study. In addition, we included 104 healthy women who were examined during the same period as a control group. Table 1 presents the baseline data of the subjects in the two groups. This study was approved by the Ethics Committee of our hospital (No. 2022-801) and strictly followed the Declaration of Helsinki. All participants provided written informed consent.

**Table 1:** Baseline data of the study subjects.

	Age	Body mass <br>index <br>(kg/m^2^)	Family history<br>of disease	Pathologic <br>Staging	Degree of <br>differentiation	Diameter of <br>tumour (cm)	Triple-negative <br>Breast Cancer
			Yes/no	I/II/III	Medium -<br>high/low	<3/≥3	Yes/no
Control group <br>(n = 104)	47.29±7.27	21.43±1.18	5/99	-	-	-	-
BC patients<br>(n = 121)	47.25±6.85	21.30±1.21	7/114	84/27/10	93/28	85/36	13/108
t	0.043	1.706	0.106	-	-	-	-
*p*	0.966	0.089	0.745	-	-	-	-

Inclusion criteria:

- (1) Pathologically confirmed BC, with the pathological type being invasive carcinoma and unilateral lesions.
- (2) Complete medical records available.
- (3) An ultrasound examination was completed at our institution.

Exclusion criteria:

- (1) Pregnant or lactating females;
- (2) Individuals with severe organic pathologies;
- (3) Patients who had undergone surgery, radiotherapy, chemotherapy, or immunotherapy prior to hospital admission;
- (4) Those suffering from acute or chronic infectious diseases;
- (5) Cases complicated by primary malignant tumours in other systems;
- (6) Patients with concurrent mental system disorders;
- (7) Subjects with haematological system diseases.

### Laboratory tests

Upon admission, fasting venous blood samples were collected from BC patients and the control group (blood samples collected within 24 hours after admission). These samples were then centrifuged at a speed of 3000 revolutions per minute (r/min) for 10 minutes in a high-speed centrifuge to isolate the serum (the serum was stored at -80°C and the test was completed within 2 weeks). Subsequently, the enzyme-linked immunosorbent assay (ELISA) technique was utilised to quantify the levels of CXCL8. The Human CXCL8/IL-8 ELISA kit (item number AJ-IL8-H) from AmyJet Scientific Inc., calibrated with the WHO standard (NIBSC 89/520), was used with intra- and inter-assay coefficients of variation <5% and <8%, respectively. In addition, we used a fully automated chemiluminescence immunoassay analyser (Myriad, CL-1000i) to detect the levels of tumour markers carcinoembryonic antigen (CEA), carbohydrate antigen (CA) 153, and CA27.29 in patients' serum.

### Statistical analysis

Two independent investigators performed data analysis in a blinded manner, and data entry was separated from statistics to avoid bias. All statistical analyses were executed using SPSS 25.0 software. The distribution of quantitative data was assessed using the Shapiro-Wilk test. For normally distributed data, expressed as mean ± standard deviation (χ̄±s), comparisons were made using the independent samples t-test. In the case of non-normally distributed data expressed as median (interquartile range), comparisons were made using the Mann-Whitney U test for non-parametric data. Qualitative data [n (%)] were compared using the chi-square test. Correlations were analysed using Pearson's correlation coefficients. Diagnostic value was evaluated using receiver operating characteristic (ROC) curve analysis. Additionally, logistic regression analysis was performed to identify related factors. Statistical significance was determined at a P-value less than 0.05.

## Results

### Comparison of CXCL8

Shapiro-Wilk test showed that CEA, CA153, and CA27.29 were normally distributed (P>0.05). Compared with the control group, CEA, CA153, and CA27.29 were elevated in BC patients as expected (*P*<0.001). In addition, CXCL8 was also higher in BC patients than in the control group (*P*<0.001), suggesting that CXCL8 may be involved in the onset and development of BC (Table 2).

**Table 2:** Comparison of CXCL8 and tumour markers in BC patients and the control group.

	CXCL8 (ng/mL)	CEA (ng/mL)	CA153 (U/mL)	CA27.29 (U/mL)
Control group (n = 104)	52.23±16.75	4.36±1.53	22.13±6.97	36.15±9.63
BC patients (n=121)	79.70±10.36	6.72±2.22	41.57±5.55	62.57±8.25
t	14.821	9.663	23.284	22.160
*p*	<0.001	<0.001	<0.001	<0.001

### Correlation between CXCL8 and tumour marker

**Figure 2:**
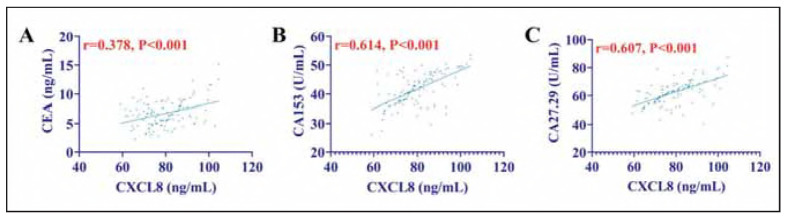
Correlation between CXCL8 and tumour marker. (A) Correlation between CXCL8 and CEA. (B) Correlation between CXCL8 and CA153. (C) Correlation between CXCL8 and CA27.29.

Pearson's correlation coefficient showed that CXCL8 and tumour markers (CEA, CA153, CA27.29) were positively correlated (*P*<0.05) in bC patients. That is, the higher the level of CXCL8, the higher the level of tumour markers (Figure 2).

### Correlation between CXCL8 and ALNM

Post-hoc power analysis (α = 0.05, effect size=0.8) showed that the test power of the current sample size (n = 36) was 0.85, which met the minimum requirements (>0.8). Compared to patients with ALNM, those without metastasis demonstrated lower levels of CXCL8 (*P*<0.001), suggesting a potential link between elevated CXCL8 and ALNM. In addition, we observed that CEA, CA153 and CA27.29 were also higher in patients with ALNM than in patients without metastasis (*P*<0.05) (Table 3).

**Table 3:** Correlation between CXCL8 and ALNM.

	n	CXCL8 (ng/mL)	CEA (ng/mL)	CA153 (U/mL)	CA27.29 (U/mL)
Not metastasised	85	77.48±9.98	6.46±1.89	40.41±5.41	60.92±6.29
Metastasised	36	84.94±9.44	7.33±2.79	44.31±4.93	66.47±10.78
t		3.821	1.992	3.716	3.543
*p*		<0.001	0.049	<0.001	<0.001

### Diagnostic effect of CXCL8 on ALNM

**Figure 3:**
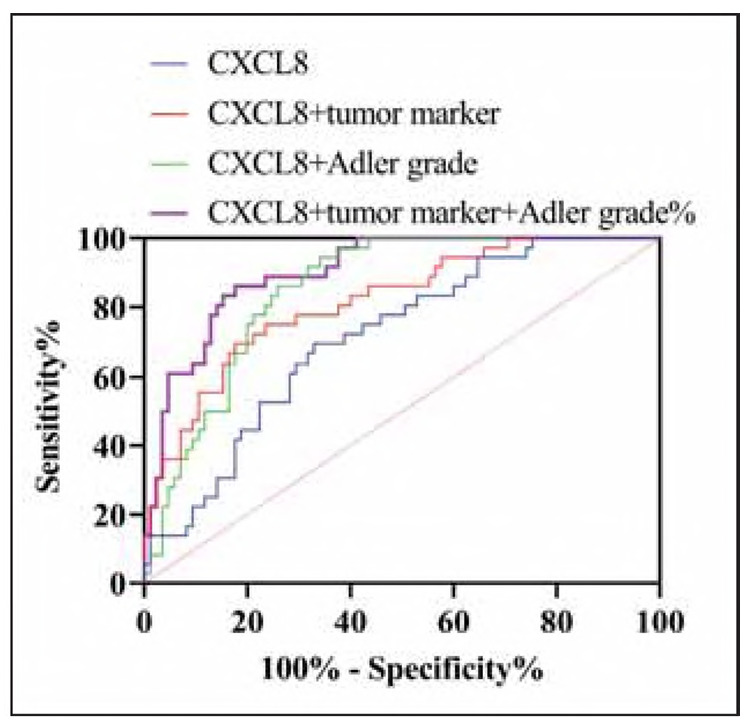
ROC curve for CXCL8 diagnosis of ALNM.

Logistic regression analysis showed that CXCL8 (β = 1.32, *P*<0.001), Adler grade (β=0.89, *P*=0.003) and CA153 (β = 0.75, *P*=0.012) contributed significantly to the model. As shown in Table 4 and Figure 3, when CXCL8 was used to diagnose BC ALNM, the area under the curve (AUC) of the ROC curve was 0.712, and when CXCL8 was combined with tumor markers or Adler grade, the AUC of the ROC curve for ALNM diagnosis was improved (0.816 and 0.852). And when CXCL8+Adler grade+tumor markers were used for the combined test, the diagnostic sensitivity for ALNM occurring in BC reached 86.11%, and the specificity reached 82.35% (*P*<0.001). The AUC of this curve was 0.903, which was superior to any other protocol.

**Table 4:** Diagnostic effect of CXCL8 on ALNM. Note: Area under the curve (AUC), 95% Confidence interval (95% CI).

	AUC	95%CI	P	Cut-off	Sensitivity%	Specificity%
CXCL8	0.712	0.617-0.806	<0.001	>80.33 ng/mL	69.44	67.06
CXCL8+tumor<br>marker	0.816	0.735-0.897	<0.001	>0.348	69.44	82.35
CXCL8+Adler<br>grade	0.852	0.786-0.917	<0.001	>.0197	94.44	65.88
CXCL8+tumor<br>marker+Adler	0.903	0.850-0.957	<0.001	>0.299	86.11	82.35

## Discussion

In this study, we found that CXCL8 was associated with the onset and progression of BC and demonstrated an excellent assessment of ALNM. These novel findings not only offer innovative perspectives and valuable references to the comprehensive evaluation of the disease state in BC patients but also establish a solid and reliable foundation for enhancing the prognosis of BC patients.

First, we found that CXCL8 levels were elevated in all BC patients compared to controls, confirming the close relationship between CXCL8 and BC progression. CXCL8, recognised as an angiogenic factor, promotes angiogenesis by binding to CXC chemokine receptors 1 and 2 to initiate a series of biological activities. Specifically, it stimulates neutrophil aggregation, which in turn releases additional CXCL8 to amplify the cascade effect, thus forming an inflammatory microenvironment that is conducive to tumour growth [9]. CXCL8 has also been found to suppress the activity of T cells, enabling tumour cells to escape immune surveillance and metastasise [12]. Subsequently, we found elevated serum levels of CXCL8 in patients with lymph node metastases, confirming that CXCL8 is also involved in the progression of lymph node metastases. CXCL8 enhances the adhesion of tumour cells to lymphatic endothelial cells by activating the NF-κB pathway and up-regulating MMP-9 to promote the degradation of extracellular matrix [8]. In other words, high CXCL8 expression may provide a new strategy for combining anti-EGFR or CXCR1/2 inhibitors, which is worthy of further exploration.

In addition, the tumour markers CEA, CA153, and CA27.29 were also significantly elevated in BC patients, as expected. Still, the trend of CEA was not as significant as that of CA153 and CA27.29, which was attributed to the lower specificity of CEA, which was elevated in only 20-30% of patients with BC. It was more commonly used for colorectal cancer surveillance [13]. The close correlation shown between CXCL8 and these tumour markers also reaffirms the potential of CXCL8 as a clinical indicator for BC. It is worth noting that although tumour markers are considered to be the most sensitive blood indicators of tumour progression, there have been many studies confirming that tumour markers are abnormally altered because of non-tumour factors [14] [15]. In comparison, CXCL8 seems to possess a more significant specificity advantage.

Certainly, single tumour markers or imaging parameters have limitations in ALNM prediction. For example, ultrasound is insufficiently sensitive for micrometastasis [16], and IL-6, although correlated with metastasis, is susceptible to interference by systemic inflammatory status [17]. In this study, we found that CXCL8+tumor markers+Adler grade can significantly improve the AUC value for ALNM prediction. The advantages are summarised in the following aspects: (1) Complementarity: CXCL8 reflect pro-metastatic activity at the molecular level, while ultrasound provides real-time information on vascular functional status, and the combination of the two can comprehensively assess tumour invasive potential. (2) Dynamic monitoring: The levels of CXCL8 and tumour markers can change with treatment, and the combination of ultrasound can dynamically adjust the prediction model and provide a basis for postoperative follow-up. (3) Clinical operability. Blood tests and ultrasound are non-invasive, and CXCL8 ELISA detection is low-cost and does not require special equipment, which is suitable for promotion in primary medical institutions.

However, the relatively small number of cases is an undeniable limitation of this study. Also, our inclusion criteria were limited to unilateral lesions, which may limit the generalizability of the results to multifocal tumours. To address this issue, it is imperative to collaborate with multiple hospitals to conduct clinical analyses with a large sample size, thereby enhancing the representativeness and comprehensiveness of the research findings. Additionally, the evaluation of the clinical efficacy and prognosis of BC using the combination of ultrasound and CXCL8 is also a key aspect worthy of consideration. We intend to systematically explore and incorporate these aspects into our subsequent research endeavours.

## Conclusion

CXCL8 is deeply involved in the process of BC ALNM by regulating the inflammatory and immune balance of the tumour microenvironment. CXCL8 combined application with ultrasound and tumour markers not only breaks through the limitations of a single technology, but also provides new ideas for the construction of individualised prediction models. In the future, longitudinal monitoring of CXCL8 levels is needed to evaluate its potential as a marker of treatment response.

## Dodatak

### Availability of data and materials

Data are available upon request.

### Funding

No funds, grants, or other support were received.

### Acknowledgements

Not applicable.

### Conflict of interest statement

All the authors declare that they have no conflict of interest in this work.
